# Ecotoxicology inside the gut: impact of heavy metals on the mouse microbiome

**DOI:** 10.1186/2050-6511-14-62

**Published:** 2013-12-11

**Authors:** Jérôme Breton, Sébastien Massart, Peter Vandamme, Evie De Brandt, Bruno Pot, Benoît Foligné

**Affiliations:** 1 Bactéries Lactiques & Immunité des Muqueuses, Centre d‘Infection et d’Immunité de Lille, Institut Pasteur de Lille, U1019, UMR 8204, Université Lille Nord de France, 1 rue du Pr Calmette, Lille cedex, BP 245, F-59019, France; 2 DNAVision SA, avenue George Lemaitre 25, Charleroi B-6041, Belgium; 3 Laboratory of Microbiology, Faculty of Sciences, Ledeganckstraat 35, Ghent B-9000, Belgium

**Keywords:** Heavy metal exposure, Gut microbiota, Mice, 16S pyrosequencing, *Turicibacter*, Denaturing gradient gel electrophoresis (DGGE)

## Abstract

**Background:**

The gut microbiota is critical for intestinal homeostasis. Recent studies have revealed the links between different types of dysbiosis and diseases inside and outside the intestine. Environmental exposure to pollutants (such as heavy metals) can also impair various physiological functions for good health. Here, we studied the impact of up to 8 weeks of oral lead and cadmium ingestion on the composition of the murine intestinal microbiome.

**Results:**

Pyrosequencing of 16S RNA sequences revealed minor but specific changes in bacterial commensal communities (at both family and genus levels) following oral exposure to the heavy metals, with notably low numbers of *Lachnospiraceae* and high numbers levels of *Lactobacillaceae* and *Erysipelotrichaceacae* (mainly due to changes in *Turicibacter spp),* relative to control animals.

**Conclusions:**

Non-absorbed heavy metals have a direct impact on the gut microbiota. In turn, this may impact the alimentary tract and overall gut homeostasis. Our results may enable more accurate assessment of the risk of intestinal disease associated with heavy metal ingestion.

## Background

Chronic ingestion of environmental heavy metals (HMs, such as lead (Pb) and cadmium (Cd)) is associated with the occurrence of various diseases. The underlying mechanism is thought to be related to excessive local and systemic oxidative stress or deregulation of immune responses. Intestinal absorption of HMs leads to accumulation in specific target organs, with severe detrimental effects on human health. However, high concentrations of non-absorbed HMs remain in the gut microenvironment, where they may have a direct impact on the gut ecosystem and its overall physiology [1,2]. The gut microbiota has been described as a complex “hidden” organ, which plays a key role in the maintenance of health; hence, the presence or absence of specific species can be essential for maintaining homeostasis both inside and outside the intestinal tract [3,4].

The gastrointestinal epithelium has several essential functions: constituting a physical barrier, ensuring mucosal immune responses and excluding or detoxifying harmful intestinal content. These processes are highly influenced by the microbiota via a complex interplay with the host [5-7]. Disturbance of the microbiota (dysbiosis) is associated with an increased risk of developing inflammatory diseases, allergic diseases and metabolic disorders; hence, it is of the utmost importance to understand microbiotal variability if we are to better understand disease states [8,9]. The most studied factors affecting microbiota composition are age, genetic background, diet and antibiotic consumption [10]. It has also been postulated that exposure to xenobiotic agents from the environment is an important factor shaping the gut microbiota. However, little attention has been given to the potential impact of bioavailable HMs on the commensal microbiota and intestinal homeostasis. We thus sought to characterize possible impact of environmental Pb and Cd on the microbial ecosystem in mice, in order to better understand the potential role of environmental factors in the etiology and pathogenesis of gastrointestinal disorders in humans.

## Methods

### Animals and ethics statement

Twenty-five Balb/C female mice (aged 6 weeks on arrival) were obtained from Charles River (Saint-Germain-sur-l’Arbresle, France). The animals were randomly divided into groups of five and housed in a controlled environment (a temperature of 22°C, a 12 h/12 h light/dark cycle and with *ad libitum* access to food and water). All animal experiments were performed according to the guidelines of the Institut Pasteur de Lille Animal Care and Use Committee and in compliance with the Amsterdam Protocol on Animal Protection and Welfare and the Directive 86/609/EEC on the Protection of Animals Used for Experimental and Other Scientific Purposes (updated in the Council of Europe’s Appendix A). The animal work was also compliant with French legislation (the French Act 87–848, dated 19-10-1987) and the European Communities Amendment of Cruelty to Animals Act 1976. The study’s objectives and procedures were approved by the Ethic and Welfare Committee for Experiments on Animals in France’s Nord-Pas-de-Calais region (approval number: 04/2011).

### Animal exposure procedures and experimental set-up

Mice were exposed to doses of either Cd (20 or 100 ppm) or Pb (100 or 500 ppm), where ppm correspond to mg L^-1^. The metals were administered continuously for 8 weeks by spiking the animals’ drinking water with CdCl_2_ or PbCl_2_ solution, as previously described [11]. In order to cover both “environmentally relevant (low)” and “critical” doses of Cd exposure and to mimic Pb poisoning, the HM doses were selected according to the respective “lowest observed adverse effect” level (LOAEL) for chronic exposure in rodents. Control animals received water with no added CdCl_2_ or PbCl_2_. Fecal pellets and cecal content were collected in tubes and weighed. Samples were snap-frozen and then stored at −80°C until nucleic acid extraction was performed, as described previously [12].

### DNA extraction and PCR amplification

16S rRNA genes were amplified using the PCR primers [13], which target the V5 and V6 hypervariable regions. The forward primer contained the sequence of the Titanium A adaptor (5′-CCATCTCATCCCTGCGTGTCTCCGACTCAG-3′) and a barcode sequence. The reverse primer contained the sequence of Titanium B adaptor primer B: (5′-CCTATCCCCTGTGTGCCTTG-3′). For each sample, a PCR mix of 100 μL contained 1 × PCR buffer, 2 U of KAPA HiFi Hotstart polymerase blend and dNTPs (Kapabiosystems, Clinisciences, Naterre, France), 300 nM primers (Eurogentec, Liège, Belgium), and 60 ng per g DNA. Thermal cycling consisted of initial denaturation at 95°C for 5 min, followed by 25 cycles of denaturation at 98°C for 20 s, annealing at 56°C for 40 s and extension at 72°C for 20 s, plus final extension at 72°C for 5 min. Amplicons were visualized on 1% agarose. Gels were stained with GelGreen Nucleic Acid gel stain in 1x Tris-acetate-EDTA (TAE) buffer and then cleaned with Wizard SV Gel and PCR Clean-up System (Promega, Charbonnieres les Bains, France), according to the manufacturer’s instructions.

### Amplicon quantitation, pooling, and pyrosequencing

Amplicon DNA concentrations were determined using the Quant-iT PicoGreen dsDNA reagent and kit (Life Tech, Carlsbad, CA) following the manufacturer’s instructions. Assays were carried out using 2 μL of cleaned PCR product in a total reaction volume of 200 μL in black, 96-well microtiter plates. Following quantitation, cleaned amplicons were combined in equimolar ratios in a single tube .The final pool of DNA was eluted in 100 μL of nuclease-free water and purified using an Agencourt Ampure XP Purification Systems, according to the manufacturer’s instructions (Agencourt Biosciences Corporation-Beckman Coulter, Beverly, MA) and then resuspended in 100 μL of TAE 1x. The concentration of the purified pooled DNA was determined using the Quant-iT PicoGreen dsDNA reagent and kit (Life Tech, Carlsbad, CA), according to the manufacturer’s instructions. Pyrosequencing was carried out using primer A on a 454 Life Sciences Genome Sequencer FLX instrument (Roche, Branford, CT) following titanium chemistry.

### 16S rRNA data analysis

The sequences were assigned to samples as a function of their sample-specific barcodes. The sequences were then checked for the following criteria [14]: (i) an almost perfect match with the barcode and primers; (ii) at least 240 nucleotides in length (not including barcodes and primers); and (iii) no more than two undetermined bases (denoted by N). By “an almost perfect match”, we mean that one mismatch/deletion/insertion per barcode or per primer was allowed. Each pyrosequenced dataset that passed quality control was assigned to a family with the RDP classifier (version 2.1, http://rdp.cme.msu.edu) with a confidence threshold > 80%. The Chao richness estimate was calculated with the Mothur software package (for more details, see http://www.mothur.org/wiki/Chao).

### Denaturing gradient gel electrophoresis (DGGE)

The variable V3 region of the 16S rRNA gene was amplified using the universal bacterial primers F357-GC and R518 [15,16]. The PCR and temperature program have been described elsewhere [17]. The resulting 16S rRNA amplicons were analyzed by DGGE fingerprinting analysis (the D-Code System from Bio-Rad, Nazareth, Belgium) using 35% to 70% denaturing gels, as previously described [16]. Each lane received 30 μl of PCR product and electrophoresis was performed at 70 V for 990 min. Next, the DGGE gels were stained for 30 min with 1 X SYBR Gold nucleic acid gel stain (S-11494; Invitrogen, Merelbeke, Belgium) in 1 X TAE buffer (Bio-Rad), and the band profiles were digitized and visualized with a charge-coupled device (CCD) camera and Quantity One software (Bio-Rad). Every fifth or sixth lane contains a reference sample (containing the V3-16S rRNA amplicons of a taxonomically well-characterized strain for each of 12 bacterial species) and fingerprint profiles were normalized using BioNumerics software (version 5.10, Applied Maths, Sint-Martens-Latem, Belgium).

### Statistics and data analysis

All statistical analyses were performed by comparing experimental groups with the control group. A non-parametric one–way analysis of variance, Mann–Whitney *U*-tests or Student’s t tests were used as appropriate. Bacterial count data are presented as the mean ± standard error of the mean (SEM). The threshold for statistical significance was set to p < 0.05.

## Results and discussion

In the present study, groups of wild-type mice underwent up to of 8 weeks continuous exposure to CdCl_2_ (20 or 100 ppm) or PbCl_2_ (100 or 500 ppm) administered in their drinking water. In an earlier study, these HM levels were sub-toxic and not associated with hepatotoxicity or changes in behavior, organ weights (liver, spleen and kidneys), body weight or overall growth (when compared with regular water-treated mice, [11]. Furthermore, none of the HM treatments had a detectable impact on our animals’ food intake, stool consistency or gut motility. Indeed, this was demonstrated by providing oral exogenous food-grade microorganisms (such as yeasts and lactic bacteria) as feces markers. All the animals exhibited similar transit times and persistence parameters (data not shown).

**Figure 1:**
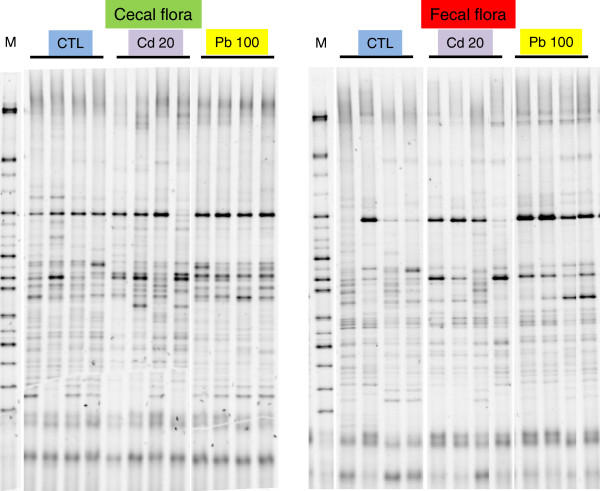
**DGGE profiles revealed microbial diversity in the cecum content and fecal pellets of mice exposed for 8 weeks to Cd and Pb salts via their drinking water.** The figure shows DGGE gels of the V5-V6 hypervariable 16S rDNA region, illustrating the microbiota’s composition in the cecum and the feces of 4 mice treated (or not) with 20 mg L^-1^ (ppm) of Cd or 100 mg L^-1^ (ppm) of Pb.

**Figure 2:**
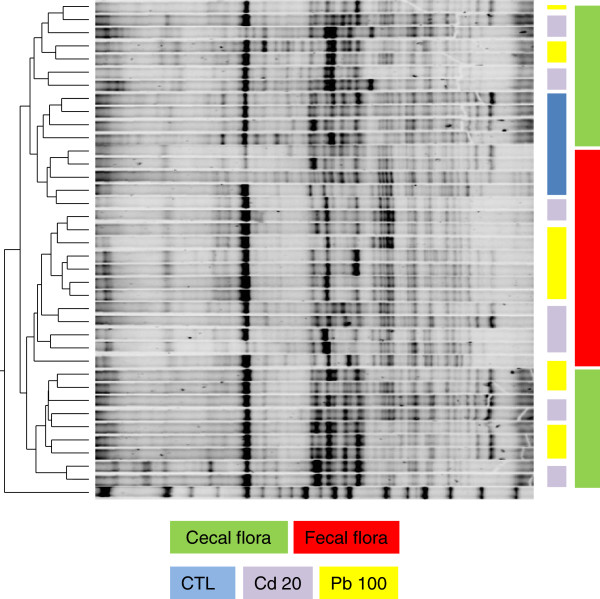
**An unweighted pair group method with arithmetic mean tree of the same gels.** Pairwise similarities were calculated with BioNumerics software (version 6.6.4), using a Dice coefficient with 0% optimization, 0.3664% fixed tolerance, exclusion of uncertain bands and no relaxed doublet matching, fuzzy logic or area sensitivity.

We measured the microbial communities’ profiles in feces and cecal content. On the basis of the DGGE results, an 8-week treatment with either Cd or Pb did not significantly modify the murine microbiota at either sampling site (Figure 1). A discriminant analysis of band classes (performed with Bionumerics software) enabled us to distinguish between fecal and colonic samples (Figure 2) and between control samples and HM-treated samples but did not pinpoint systematic differences between Pb and Cd treatments or between low and high concentrations of the HMs (results not shown). This finding contrasts with a recent report in which oral Cd had harmful effects on the viability of some components of the mouse microbiota [18]. This disparity might be explained by the fact that Fazeli and coworkers used restrictive conventional culture methods, whereas we used a molecular approach.

**Figure 3:**
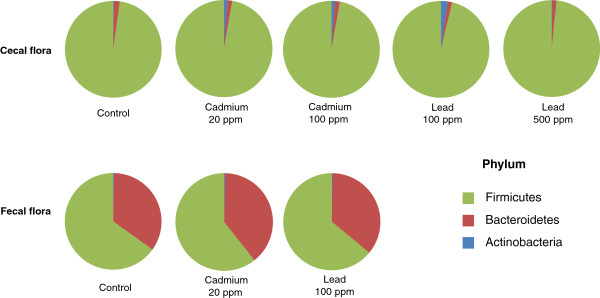
**Distribution of bacterial phylotypes in the cecum content and fecal pellets of mice exposed for 8 weeks to Cd (20 or 100 ppm) or Pb (100 or 500 ppm) salts via their drinking water.** 16S rRNA-base analyses were derived from 454/Roche multitag pyrosequencing. Data are expressed as the mean percentage abundance of the total assignment (n = 5 animals per group). In line with the literature data, most of the bacteria in untreated (control) mice belonged to Firmicutes or the Bacteroidetes, whereas Actinobacteria were very rare.

**Figure 4:**
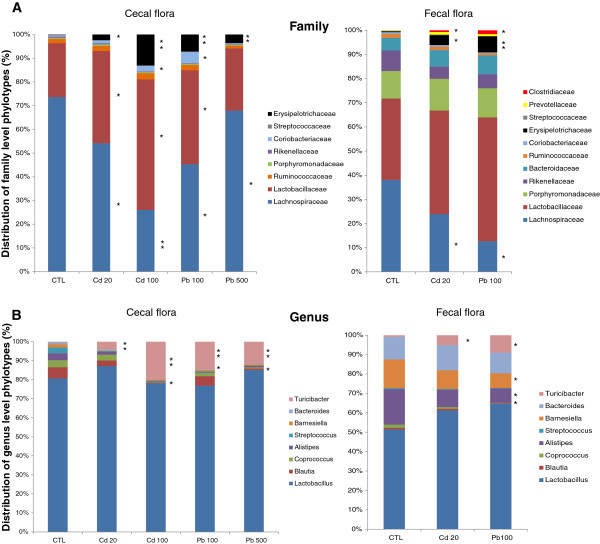
**Distribution of bacterial subgroups in the cecum content and fecal pellets of mice exposed for 8 weeks to Cd (20 or 100 mg L**^**-1**^**) or Pb (100 or 500 mg L**^**-1**^**) salts via their drinking water. (A)** Family-level and **(B)** genus-level. 16S rRNA-base analyses were derived from 454/Roche multitag pyrosequencing. Data are expressed as the mean percentage abundance (n = 5 animals per group). Only operational taxonomic units (OTU’s) present in dominant families (> 0.1%) were considered. *: p < 0.05 and **: p < 0.01: significantly different from the control group (water with no added Cd or Pb) for the corresponding taxa. The color code is defined in the inset on the right of the Figure.

A more in-depth analysis of the cecal and fecal microbiome was carried out via 454 pyrosequencing of the V5-V6 region of the 16S rRNA (Table 1, Figures 3 and 4). We generated a dataset consisting of 197,143 filtered, high-quality 16S rRNA gene sequences (mean ± SD number of sequences per sample: 11,596 ± 6060). With operational taxonomic unit (OTU) cut-offs of 0.03, 0.05 or 0.10, the samples from the Cd, Pb and control groups did not differ significantly in terms of microbial richness (as estimated by the Chao richness index) or biodiversity (assessed by a nonparametric Shannon index). With an OTU cut-off of 0.03, the mean number of clusters was 1244 ± 381. The abundance of the two major phyla (the Firmicutes and Bacteroidetes) was similar in all three groups, whereas there were few Actinobacteria (Figure 3). In contrast, treatment with the two HMs was associated with a change in the composition of the colonic microbiota at both the family and genus levels. In fact, eight weeks of oral Cd or Pb treatment caused small but statistically significant differences in numbers of Prevotellaceae and Clostridiaceae (especially in the feces). Significant differences (p < 0.05) in the relative abundance of several other families in both cecal and fecal samples were observed, with low numbers of Lachnospiraceae and high numbers of Lactobacillaceae and Erysipelotrichaceae in the HM-treated groups (Figure 4A). Within the Erysipelotrichaceae family, numbers of *Turicibacter* (Figure 4B), coprococci, streptococci, *Blautia, Barneselia* and *Allistipes* were higher in HM-treated groups than in controls. In general, we observed lower genus diversity in the HM-treated groups. Low bacterial diversity and low number of Lachnospiraceae have been linked to intestinal inflammation and considered as a predisposition to colitis [19,20]. Whether changes in Lachnospiraceae, Lactobacillaceae and Erysipelotrichaceae are consistently linked with inflammation remains to be established. However, the frequent literature reports on changes in the abundance of these groups in the mouse microbiome indicate that these groups are more sensitive to external factors than other, less abundant groups are. However, cautious interpretation is necessary because of the low family-level resolution of metagenomics, which prevents reliable microbial community analyses under in inflammatory conditions, for example [21].

**Table 1:** Relative distributions of bacterial phylotypes, families and genera in (i) the cecum content of mice orally exposed for 8 weeks to Cd (20 or 100 ppm) or Pb (100 or 500 ppm) salts and (ii) the fecal pellets for mice orally exposed for 8 weeks to Cd (20 ppm) or Pb (100 ppm)

**Phylum**	**Cd0Pb0**	**Cd20**	**Cd100**	**Pb100**	**Pb500**
**Cecal content**					
**Actinobacteria**	0.25%	0.59%	1.19%	2.27%	0.25%
(range)	(0.11-0.40)	(0.16-1.30)	(0.36-1.84)	(0.11-5.39)	(0.10-0.40)
SEM	0.049	0.210	0.272	1.113	0.161
*P value*	*-*	*0.0775*	*0.0045*	*0.0537*	*0.478*
**Bacteroidetes**	1.9%	2.58%	1.60%	1.47%	1.36%
(range)	s(1.29-2.28)	(0.35-7.66)	(0.31-4.97)	(0.30-3.36)	(0.8-2.40)
SEM	0.183	1.328	0.854	0.612	0.3
*P value*	*-*	*0.3131*	*0.3697*	*0.2578*	*0.0869*
**Firmicutes**	97.8%	96.77%	97.2%	96.24%	98.36%
(range)	(97.4-98.4)	(92.0-99.3)	(93.1-98.8)	(91.1-99.6)	(97.9-99.0)
SEM	0.187	1.297	1.037	1.739	0.272
* P value*	*-*	*0.2269*	*0.2916*	*0.1986*	*0.1755*
**Fecal pellet**					
**Actinobacteria**	0.30%	0.39%		0.24%	
(range)	(0.18-0.50)	(0.13-0.65)		(0.08-0.42)	
SEM	0.053	0.089		0.058	
*P value*	*-*	*0.2304*		*0.2188*	
**Bacteroidetes**	34.4%	38.8%		35.65%	
(range)	(12.5-50.8)	(30.1-7.5)		(22.4-51.5)	
SEM	6.56	10.84		8.65	
*P value*	*-*	*0.3670*		*0.4553*	
**Firmicutes**	64.7%	60.44%		63.85%	
(range)	(48.2-71.3)	(47.5-90.9)		(38.2-77.5)	
SEM	6.73	10.86		8.72	
*P value*	*-*	*0.3736*		*0.4701*	
**Family**	**Cd0Pb0**	**Cd20**	**Cd100**	**Pb100**	**Pb500**
**Cecal content**					
***Lachnospiraceae***	72.6%	53.17%	25.9%	43.7%	67.5%
(range)	(33.2-88.3)	(26.8-76.6)	(10.3-28.5)	(27.1-61.4)	(59.1-75.0)
SEM	10.01	9.24	4.15	6.09	2.62
*P value*	*-*	*0.037*	*0.0081*	*0.0378*	*0.05859*
***Lactobacillaceae***	22.34%	38.20%	54.68%	38.24%	26.1%
(range)	(5.6-64.9)	(19.01-60.6)	(41.6-81.6)	(28.7-67.1)	(19.2-32.1)
SEM	10.88	8.54	7.06	7.26	2.09
*P value*	*-*	*0.05859*	*0.0379*	*0.0379*	*0.05859*
***Ruminococcaceae***	1.82%	2.15%	2.64%	2.15%	1.14%
(range)	(0.72-3.01)	(0.76-5.91)	(0.81-7.21)	(0.63-3.22)	(0.58-1.67)
SEM	0.442	0.836	0.876	0.934	0.939
*P value*	*-*	*0.3804*	*0.2627*	*0.3106*	*0.0997*
***Porphyromonadaceae***	0.42%	0.63%	0.42%	0.49%	0.41%
(range)	(0.0-1.25)	(0.0-2.24)	(0.0-1.41)	(0.0-1.65)	(0.05-1.24)
SEM	0.228	0.438	0.433	0.415	0.411
*P value*	*-*	*0.3152*	*0.4774*	*0.4139*	*0.0641*
***Rikenellaceae***	0.51%	0.42%	0.27%	0.21%	0.26%
(range)	(0.0-1.32)	(0.0-1.32)	(0.0-0.92)	(0.0-0.6)	
(0.0-0.47)					
SEM	0.250	0.245	0.259	0.174	0.174
*P value*	*-*	*0.3883*	*0.2216*	*0.1564*	*0.1800*
***Coriobacteriaceae***	0.49%	1.19%	2.38%	4.57%	0.36%
(range)	(0.2-0.76)	(0.23-2.6)	(0.69-4.35)	(0.2-11.75)	(0.1-0.51)
SEM	0.111	0.0939	0.107	0.433	0.138
*P value*	*-*	*0.1736*	*0.01414*	*0.0250*	*0.3*
***Streptococcaceae***	0.3%	0.14%	0.15%	0.24%	0.04%
(range)	(0.0-0.50)	(0.05-0.3)	(0.0-0.66)	(0–0.75)	(0–0.1)
SEM	0.102	0.0239	0.0983	0.0894	0.0581
*P value*	*-*	*0.0901*	*0.1905*	*0.3711*	*0.0164*
***Erysipelotrichaceae***	0.1%	2.32%	12.98%	6.89%	3.6%
(range)	(0.0-0.24)	(0.42-8.02)	(3.82-28.12)	(2.13-14)	(1.73-6.05)
SEM	0.044	0.232	0.219	0.268	0.02236
*P value*	*-*	*0.0182*	*0.0096*	*0.0096*	*0.00051*
**Family**	**Cd0Pb0**	**Cd20**		**Pb100**	
**Fecal pellet**					
***Lachnospiraceae***	37.36%	23.67%		12.55%	
(range)	(18.8-86.9)	1.77-82.9)		(3.52-26.6)	
SEM	12.81	14.14		17.28	
*P value*	*-*	*0.0453*		*0.023*	
***Lactobacillaceae***	32.99%	42.77%		50.88%	
(range)	(10.3-51.2)	(4.49-67.1)		(25.1-65.6)	
SEM	8.58	8.87		10.02	
*P value*	*-*	*0.2782*		*0.1121*	
***Ruminococcaceae***	1.70%	1.4%		0.83%	
(range)	(0.24-3.46)	(0.08-4.58)		(0.11-1.61)	
SEM	0.65	0.65		0.86	
*P value*	*-*	*0.3916*		*0.1275*	
***Porphyromonadaceae***	11.14%	13.01%		12%	
(range)	(3.46-16.25)	(1.60-22.04)		(4.88-19.6)	
SEM	2.22	3.11		3.84	
*P value*	*-*	*0.3447*		*0.4075*	
***Rikenellaceae***	8.36%	4.99%		5.82%	
(range)	(5.71-14.18)	(1.93-16.07)		(0.45-18.5)	
SEM	1.92	2.47		2.87	
*P value*	*-*	*0.1736*		*0.2626*	
***Coriobacteriaceae***	0.68%	0.67%		0.41%	
(range)	(0.03-0.99)	(0.32-1.32)		(0.16-0.67)	
SEM	0.137	0.114		0.159	
*P value*	*-*	*0.4841*		*0.0677*	
***Streptococcaceae***	0.27%	0.26%		0.20%	
(range)	(0.0-0.68)	(0.02-0.69)		(0–0.36)	
SEM	0.127	0.129		0.09	
*P value*	*-*	*0.4969*		*0.3060*	
***Erysipelotrichaceae***	0.33%	4.05%		6.56%	
(range)	(0.0-0.89)	(0.48-15.53)		(0.44-20.7)	
SEM	0.158	0.399		0.4033	
*P value*	*-*	*0.0585*		*0.0107*	
***Prevotellaceae***	0.14%	1.06%		0.75%	
(range)	(0.0-0.25)	(0.08-4.14)		(0.11-1.22)	
SEM	0.058	0.806		0.812	
*P value*	*-*	*0.1345*		*0.049*	
***Clostridiaceae***	0.00%	0.675%		1.55%	
(range)	(0.0-0.00)	(0.0-2.17)		(0.0-3.06)	
SEM	0.0	0.094		0.0940	
*P value*	*-*	*0.0628*		*0.0086*	
**Genus**	**Cd0Pb0**	**Cd20**	**Cd100**	**Pb100**	**Pb500**
**Cecal content**					
***Lactobacillus***	75.28	84.57%	77.4%	74.8%	83.82%
(range)	(60–98.9)	(68–91.11)	(57.6-93.6)	(54.1-90.6)	(80.4-89.3)
SEM	7.554	4.419	5.97	7.741	1.589
*P value*	*-*	*0.2229*	*0.1248*	*0.1234*	*0.1503*
***Blautia***	5.39%	2.82%	0.36%	4.90%	0.78%
(range)	(0–12.6)	(0.45-8.8)	(0–0.97)	(0–9.2)	(0.3-1.22)
SEM	2.58	1.526	0.176	1.990	0.165
*P value*	*-*	*0.2456*	*0.1085*	*0.1136*	*0.2036*
***Coprococcus***	3.49%	3.06%	0.46%	1.65%	0.64%
(range)	(0–7.95)	(0.22-6.49)	(0.14-0.73)	(0.7-3.62)	(0.32-1.2)
SEM	1.651	1.172	0.110	0.521	0.151
*P value*	*-*	*0.1874*	*0.2086*	*0.2677*	*0.2757*
***Alistipes***	3.42%	1.40%	0.41%	0.40%	0.74%
(range)	(0.0-12.5)(0.0-4.0)	(0.0-1.49)	(0.0-1.16)	(0.0-1.34)	
SEM	2.40	0.725	0.273	0.232	0.221
*P value*	*-*	*0.2084*	*0.0439*	*0.4421*	*0.0461*
***Steptococcus***	2.84%	0.35%	0.24%	0.47%	0.11%
(range)	(0.0-6.67)	(0.11-0.80)	(0.0-1.05)	(0.0-1.45)	(0.0-0.24)
SEM	1.235	0.132	0.204	0.274	0.0514
*P value*	*-*	*0.4189*	*0.0522*	*0.1590*	*0.0616*
***Barnesiella***	1.56%	0.34%	0.44%	0.66%	0.72%
(range)	(0.0-6.67)	(0.0-0.8)	(0.0-1.35)	(0.0-2.6)	(0.0-2.27)
SEM	1.295	0.152	0.244	0.503	0.404
*P value*	*-*	*0.1598*	*0.4157*	*0.4842*	*0.1505*
***Bacteroides***	0.95%	0.44%	0.09%	0.11%	0.36%
(range)	(0.0-3.33)	(0.0-1.6)	(0.0-0.37)	(0–0.54)	(0–1.29)
SEM	0.631	0.292	0.071	0.108	0.236
*P value*	*-*	*0.0401*	*0.0356*	*0.0489*	*0.0229*
***Turicibacter***	0.28%	4.02%	19.63%	14.34%	11.32%
(range)	(0.0-1.15)	(0.76-11.8)	(4.48-41.3)	(3.09-22.15)	(6.37-15.4)
SEM	0.222	1.987	6.566	4.599	1.749
*P value*	*-*	*0.0492*	*0.0092*	*0.0078*	*0.0001*
**Genus**	**Cd0Pb0**	**Cd20**		**Pb100**	
**Fecal pellet**					
***Lactobacillus***	49.31%	58.0%		63.07%	
(range)	(8.96-73.18)	(22.4-88.4)		(34.4-82.9)	
SEM	10.71	12.14		11.67	
*P value*	*-*	*0.3032*		*0.2053*	
***Blautia***	0.73%	0.75%		0.28%	
(range)	(0.0-2.99)	(0.0-3.28)		(0.0-0.64)	
SEM	0.570	0.634		0.143	
*P value*	*-*	*0.4873*		*0.2331*	
***Coprococcus***	1.61%	0.83%		0.14%	
(range)	(0.0-5.97)	(0.0-3.48)		(0.0-0.32)	
SEM	1.101	0.664		0.065	
*P value*	*-*	*0.2795*		*0.1091*	
***Alistipes***	17.5%	8.47%		7.20%	
(range)	(7.2-29.8)	(2.0-19.3)		(0.43-24.5)	
SEM	3.871	3.822		4.454	
*P value*	*-*	*0.0678*		*0.0495*	
***Steptococcus***	0.50%	0.38%		0.28%	
(range)	(0.0-1.44)	(0.1-0.87)		(0.0-0.5)	
SEM	0.263	0.152		0.094	
*P value*	*-*	*0.3494*		*0.2254*	
***Barnesiella***	14.18%	9.13%		7.38%	
(range)	(6.8-25.3)	(5.12-16.7)		(2.4-11.8)	
SEM	3.049	2.216		1.711	
*P value*	*-*	*0.1086*		*0.0438*	
***Bacteroides***	11.17%	12.32%		10.46%	
(range)	(4.7-19.6)	(2.08-39.9)		(3.76-24.8)	
SEM	3.175	7.419		4.364	
*P value*	*-*	*0.4448*		*0.4492*	
***Turicibacter***	0.75%	4.81%		8.71%	
(range)	(0.0-1.49)	(0.82-17.6)		(2.84-28.24)	
SEM	0.307	3.236		4.996	
*P value*	*-*	*0.0483*		*0.0281*	

Data are expressed as the mean, range and SEM percentage abundance of the total assignment (n = 5 animals per group) and the corresponding p value is given in italics. The threshold for statistical significance was set to p < 0.05.

The genus *Turicibacter* was previously detected in the ileal pouch of an ulcerative colitis patient [22], in human appendicitis [23] and in infectious states in piglets. Interestingly, high levels of *Turicibacter* were observed in mice fed an iron-free diet (in which these bacteria might favor anti-inflammatory effects) [24] and in colitis-resistant CD8-knock-out mice (where it is potentially involved in the anti-inflammatory phenotype) [25]. The ongoing sequencing of several *Turicibacter* spp genomes will hopefully clarify their function as part of the microbiota and elucidate their role(s) in the interaction between HM exposure and inflammation [26]. Lastly, the lactobacilli’s apparent ability to tolerate HMs might be helpful for bioremediation purposes, since some microorganisms can bind labile metal ions and remove them from the environment [27]. In theory, HM-resistant, innocuous strains with anti-oxidant and anti-inflammatory properties could be used as probiotics by combining their chelating properties with targeted treatment of the xenobiotics’ harmful effects on the host’s microbiota [28,29].

Laboratory mice have a less complex gut microbiota than humans and there are only slight mouse-to-mouse variations when groups of individuals are housed together. Nevertheless, HM-associated differences in the microbiota were observed in all individual, exposed mice (data not shown). Our DGGE and metagenomics results confirmed a clear link between ingestion of HMs and the composition of the gut microbiota. The marked, environmentally-induced alteration in the gut microbiota also suggests a link between HM exposure and inflammation. However, the functional classification of groups of bacteria as “predisposing”, “colitogenic” or even “protective” is hotly debated and difficult to investigate [23]. Besides producing quantitative and qualitative changes in the gut microbiota, HMs also impact (directly or indirectly) intestinal homeostasis through their many local effects (on the epithelia mucosa) and systemic effects. Indeed, we previously reported that chronic ingestion of Cd and Pb induced (i) anemia and tissue iron loss from tissues, (ii) slight but consistent changes in the expression of transport-related genes, (iii) the small intestine and colon’s oxidative and inflammatory status and (iv) genotoxicity [11]. It is difficult to predict the net inflammatory balance in this context, since both harmful and adaptive events occur together. We also recently emphasized the key role of the microbiota in the process of HM absorption and dissemination throughout the body - illustrating the complex metal-microbe-host interplay that operates [30]. Our present ecotoxicological results complement that first attempt to identify the impact of HMs on the gut’s microbial ecology. This is in line with the need to develop a more comprehensive view of environmental exposure, i.e. one that is not restricted to the mere entry of xenobiotics into the body but also takes account of inflammation, oxidative stress, other gut flora, metabolic processes and a continually fluctuating chemical environment. Defining this type of integrated “exposome” may provide a way of causally linking long-term exposure to the occurrence of chronic disease [31].

## Conclusions

Non-absorbed heavy metals have a direct impact on the gut microbiota. In turn, this may impact the alimentary tract and overall gut homeostasis. Our results may enable more accurate assessment of the risk of intestinal disease associated with heavy metal ingestion. Further studies are needed to understand the complex crosstalk between the gut microbiota and the host, interpret the clinical consequences of exposure to xenobiotics and assess the relationship between the environment and disease susceptibility.

## Competing interests

None of all authors have conflicts of interest to declare.

## Authors’ contributions

BF and BP: study conception and design and drafting of the manuscript; BF, JB, SM, EDB and PVD: data acquisition; BF, SM, PVD and BP: data analysis and interpretation. All authors read and approved the final manuscript.

## Pre-publication history

The pre-publication history for this paper can be accessed here:

http://www.biomedcentral.com/2050-6511/14/62/prepub
